# Assessing the Relationship between Annual Surface Temperature Changes and the Burden of Dengue: Implications for Climate Change and Global Health Outcomes

**DOI:** 10.3390/tropicalmed8070351

**Published:** 2023-07-02

**Authors:** Oliver Mendoza-Cano, Xóchitl Trujillo, Miguel Huerta, Mónica Ríos-Silva, Agustin Lugo-Radillo, Verónica Benites-Godínez, Jaime Alberto Bricio-Barrios, Eder Fernando Ríos-Bracamontes, Juan Manuel Uribe-Ramos, Greta Mariana Baltazar-Rodríguez, Efrén Murillo-Zamora

**Affiliations:** 1 Facultad de Ingeniería Civil, Universidad de Colima, km. 9 Carretera Colima-Coquimatlán, Colima 28400, México; 2 Centro Universitario de Investigaciones Biomédicas, Universidad de Colima, Av. 25 de Julio 965, Col. Villas San Sebastián, Colima 28045, México; 3 Centro Universitario de Investigaciones Biomédicas, CONAHCyT—Universidad de Colima, Av. 25 de Julio 965, Col. Villas San Sebastián, Colima 28045, México; 4 CONAHCyT—Facultad de Medicina y Cirugía, Universidad Autónoma Benito Juárez de Oaxaca, Ex Hacienda Aguilera S/N, Carr. a San Felipe del Agua, Oaxaca 68020, México; 5 Coordinación de Educación en Salud, Instituto Mexicano del Seguro Social, Calzada del Ejercito Nacional 14, Col. Fray Junípero Serra, Nayarit 63160, México; 6 Unidad Académica de Medicina, Universidad Autónoma de Nayarit, Ciudad de la Cultura Amado Nervo, Nayarit 63155, México; 7 Facultad de Medicina, Universidad de Colima, Av. Universidad 333, Col. Las Víboras, Colima 28040, México; 8 Departamento de Medicina Interna, Hospital General de Zona No. 1, Instituto Mexicano del Seguro Social, Av. Lapislázuli 250, Col. El Haya, Colima 28984, México; 9 Escuela de Medicina y Ciencias de la Salud, Instituto Tecnológico y de Estudios Superiores de Monterrey, Campus Guadalajara, Av. General Ramón Corona No. 2514, Col Nuevo México, Jalisco 45201, México; a01635972@tec.mx; 10 Unidad de Investigación en Epidemiología Clínica, Instituto Mexicano del Seguro Social, Av. Lapislázuli 250, Col. El Haya, Colima 28984, México

**Keywords:** climate change, global warming, dengue, burden of disease

## Abstract

Dengue fever remains a significant global health concern, imposing a substantial burden on public health systems worldwide. Recent studies have suggested that climate change, specifically the increase in surface temperatures associated with global warming, may impact the transmission dynamics of dengue. This study aimed to assess the relationship between annual surface temperature changes from 1961 to 2019 and the burden of dengue in 185 countries. The dengue burden was evaluated for 2019 using disability-adjusted life years (DALYs) and the annual rate of change (ARC) in DALY rates assessed from 1990 to 2019. A cross-sectional and ecological analysis was conducted using two publicly available datasets. Regression coefficients (β) and 95% confidence intervals (CI) were used to examine the relationship between annual surface temperature changes and the burden of dengue. The results revealed a significant negative relationship between mean surface temperatures and DALY rates in 2019 (β = −16.9, 95% CI −26.9 to −6.8). Similarly, a significant negative relationship was observed between the temperature variable and the ARC (β = −0.99, 95% CI −1.66 to −0.32). These findings suggest that as temperatures continue to rise, the burden of dengue may globally decrease. The ecology of the vector and variations in seasons, precipitation patterns, and humidity levels may partially contribute to this phenomenon. Our study contributes to the expanding body of evidence regarding the potential implications of climate change for dengue dynamics. It emphasizes the critical importance of addressing climate change as a determinant of global health outcomes.

## 1. Introduction

Dengue fever is primarily transmitted by *Aedes* (*Ae.*) *aegypti* and *Ae. albopictus* mosquitoes, and remains a significant global health concern imposing a substantial burden on public health systems worldwide, with nearly 400 million dengue infections occurring yearly [1]. The incidence patterns of this vector-borne disease display heterogeneity. Despite an overall decrease in global dengue rates during the past decade, there is a persistent upward trend observed in some hyperendemic regions, as well as in regions categorized as middle and high-middle income. This trend is particularly prominent among individuals aged 14–70 years old [2,3]. Recent studies have suggested that climate change increases explicitly in surface temperatures secondary to global warming, which may have implications for dengue transmission dynamics [4,5,6,7].

Global warming is a well-documented phenomenon observed in recent decades and is primarily caused by increased concentrations of greenhouse gases (GHGs) in the Earth’s atmosphere [8]. Human activities, particularly burning fossil fuels such as coal, oil, and natural gas for energy production and transportation, are the predominant contributors to this increase [9]. These activities release carbon dioxide (CO_2_), the most abundant GHG, into the atmosphere [10]. Additionally, deforestation, land-use changes, and industrial processes release other GHGs such as methane (CH_4_) and nitrous oxide (N_2_O) [11]. These GHGs trap heat from the sun, leading to the greenhouse effect and the subsequent warming of the planet.

Understanding the potential relationship between global warming and dengue transmission is crucial for developing effective preventive and control strategies [12]. While numerous studies have investigated the influence of temperature on the occurrence and distribution of dengue, there remains a need for comprehensive analyses that encompass a large number of countries over an extended period.

This study aimed to evaluate the relationship between the annual surface temperature change from 1961 to 2019 and the burden of dengue in 185 countries. The burden of the disease was evaluated for the year 2019 (measured in disability-adjusted life years, DALYs) and from 1990 to 2019 as the annual rate of change (ARC) in the observed DALY rates. By examining long-term temperature trends and their association with the dengue burden, we aimed to contribute to the existing knowledge regarding the impact of climate change on the global distribution and dynamics of dengue.

DALYs serve as a comprehensive measure of the overall disease burden by integrating mortality and disability into a single quantifiable unit. The calculation of DALYs involves the summation of years of healthy life lost due to premature death (YLLs) and years lived with a disability (YLDs) [13]. In general terms, YLLs are calculated by multiplying the number of deaths by the standard life expectancy at the age of death. This measure quantifies the reduction in life expectancy due to a particular cause. On the other hand, YLDs are determined by multiplying the number of new cases of a disease by a disability weight, along with the average duration a person lives with the disease before remission or death. YLDs capture the diminished quality of life experienced by individuals with injuries or illnesses [14].

We hypothesized that countries experiencing higher increases in annual surface temperatures would exhibit correspondingly higher dengue rates, and higher annual rates of change in dengue burden; however, we also acknowledged that the relationship between temperature and dengue transmission is complex and influenced by various ecological, environmental, and socioeconomic factors.

The findings of this study have the potential to inform public health policies and interventions aimed at mitigating the impact of dengue fever in the face of ongoing climate change. By elucidating the relationship between temperature changes and dengue burden, we can identify vulnerable regions and populations that require targeted interventions and develop adaptive strategies to minimize the disease’s public health impact.

## 2. Materials and Methods

A cross-sectional analysis was performed using two publicly available datasets and employing an ecological (country-level) approach. Both databases were consulted on 25 May 2023. First, the annual surface temperature change from 1961 to 2021 was extracted from the Climate Change Dashboard provided by the International Monetary Fund [15]. This dataset, includes relevant macroeconomic indicators related to climate change. The analyzed dataset represents the average change in surface temperature (°C) from 1961 to 2021, using temperatures from 1951 to 1980 as the baseline. The data for 2019 were obtained; therefore, the absolute change in surface temperature in 2019, compared to the baseline, was utilized. This dataset is derived from the Goddard Institute for Space Studies Surface Temperature Analysis (GISTEMP) data provided by the National Aeronautics and Space Administration Goddard Institute for Space Studies (NASA GISS).

Furthermore, we utilized the Global Burden of Disease and Risk Factors Study 2019 (GBD 2019) to acquire the DALY rates (per 100,000) attributed to dengue in 2019, as well as the annual rate of change (ARC, %) in these rates from 1990 to 2019 [16]. The ARC incorporated in our analysis was calculated as the logarithmically transformed ratio of 2019 estimates to 1990 estimates, divided by the number of years [17].

All countries with complete data were included in the final analysis, resulting in a sample size of 185 nations. The countries were categorized into the following continents: Africa (Algeria, Angola, Benin, Botswana, Burkina Faso, Burundi, Cabo Verde, Cameroon, Central African Republic, Chad, Comoros, Democratic Republic of Congo, Republic of Congo, Côte d’Ivoire, Djibouti, Egypt, Equatorial Guinea, Eritrea, Eswatini, Ethiopia, Gabon, Gambia, Ghana, Guinea, Guinea-Bissau, Kenya, Lesotho, Liberia, Libya, Madagascar, Malawi, Mali, Mauritania, Mauritius, Morocco, Mozambique, Namibia, Niger, Nigeria, Rwanda, São Tomé and Príncipe, Senegal, Seychelles, Sierra Leone, Somalia, South Africa, South Sudan, Sudan, Tanzania, Togo, Tunisia, Uganda, Zambia, and Zimbabwe); Asia (Afghanistan, Armenia, Azerbaijan, Bahrain, Bangladesh, Bhutan, Brunei Darussalam, Cambodia, China, Georgia, India, Indonesia, Iran, Iraq, Israel, Japan, Jordan, Kazakhstan, Kuwait, Kyrgyzstan, Lao People’s Democratic Republic, Lebanon, Malaysia, Maldives, Mongolia, Myanmar, Nepal, North Korea, Oman, Pakistan, Philippines, Qatar, Russia, Saudi Arabia, Singapore, South Korea, Sri Lanka, Syrian Arab Republic, Taiwan, Tajikistan, Thailand, Timor-Leste, Turkey, Turkmenistan, United Arab Emirates, Uzbekistan, Vietnam, and Yemen); Europe (Albania, Andorra, Austria, Belarus, Belgium, Bosnia and Herzegovina, Bulgaria, Croatia, Cyprus, Czech Republic, Denmark, Estonia, Finland, France, Germany, Greece, Hungary, Iceland, Ireland, Italy, Latvia, Liechtenstein, Lithuania, Luxembourg, Malta, Moldova, Monaco, Montenegro, Netherlands, North Macedonia, Norway, Poland, Portugal, Romania, San Marino, Serbia, Slovakia, Slovenia, Spain, Sweden, Switzerland, Ukraine, and United Kingdom); America (Antigua and Barbuda, Argentina, Bahamas, Barbados, Belize, Bolivia, Brazil, Canada, Chile, Colombia, Costa Rica, Cuba, Dominica, Dominican Republic, Ecuador, El Salvador, Grenada, Guatemala, Guyana, Haiti, Honduras, Jamaica, Mexico, Nicaragua, Panama, Paraguay, Peru, Saint Kitts and Nevis, Saint Lucia, Saint Vincent and the Grenadines, Suriname, Trinidad and Tobago, United States, Uruguay, and Venezuela); and Oceania (Australia, Fiji, Kiribati, Marshall Islands, Micronesia, Nauru, New Zealand, Palau, Papua New Guinea, Samoa, Solomon Islands, Tonga, Tuvalu, and Vanuatu).

We calculated summary statistics and employed linear regression models to determine regression coefficients (β) and 95% confidence intervals (CI). Two models were constructed: one to assess the association between annual surface temperature change and the DALY rate (in 2019), and another to examine the relationship between temperature change and the ARC (1990–2019) in DALY rates.

Since we analyzed ecological variables from publicly available datasets, the requirement for approval and ethical considerations in health research was waived. Nonetheless, the research group followed rigorous ethical guidelines.

## 3. Results

**Figure 1:**
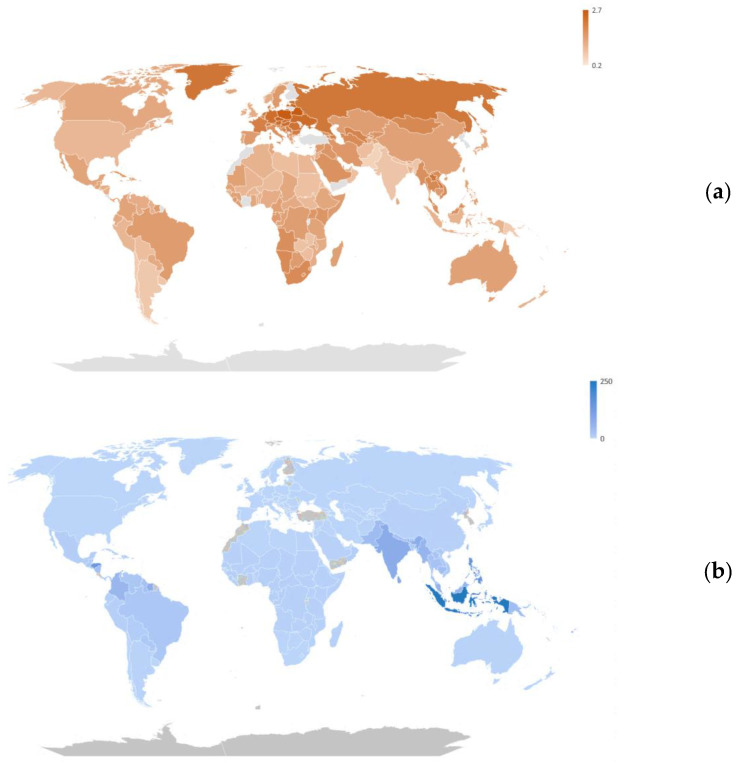

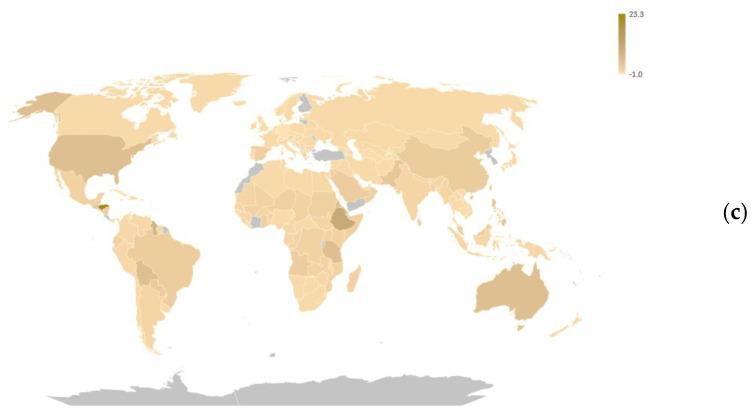
Annual surface temperature (°C) change from 1961 to 2021 (**a**); DALY rates (per 100,000) attributed to dengue in 2019 (**b**); and annual rate of change (%) in DALY rates from 1990 to 2019 (**c**).

All countries analyzed demonstrated a significant increase in mean surface temperatures in 2019 compared to the baseline assessment. As illustrated in Figure 1a, the extent of this increase varied among countries, with the Cook Islands observing the slightest rise of 0.2 °C, while Belarus recorded the highest increase of 2.7 °C. The median temperature increase across all countries was 1.4 °C, suggesting a consistent upward trend in global temperatures.

Figure 1b illustrates the DALY rates in 2019. The analyzed countries showed variations in DALY rates, with a median rate of 7.0 per 100,000. The range of DALY rates was substantial, spanning from 0.1 (Spain) to 250 (Indonesia) per 100,000. Alongside Indonesia, several other countries documented high DALY rates, including Tonga (177), the Philippines (158), Maldives (148), and Honduras (143 per 100,000), as depicted in Figure 1b.

Heterogeneous trends in DALY rates were observed from 1990 to 2019. While 63 countries displayed no change in rates during this interval, 22 nations experienced a notable decreasing ARC. The median estimate for these countries was −0.3%, with ARC ranging from −1.0% to −0.1%. In contrast, the remaining countries demonstrated increasing trends, with a median ARC of 1.0%. The range of the ARC in these countries varied from 0.1% to 23.3%, exemplified by Vietnam and Honduras, respectively. These trends are graphically presented in Figure 1c, highlighting the diverse trajectories of DALY rates across countries.

The linear regression models revealed a significant and negative relationship be-tween mean surface temperatures and DALY rates in 2019 (β = −16.9, 95% CI −26.9 to −6.8, p = 0.001). This suggests that higher temperatures are associated with lower DALY rates. Similarly, the relationship between the temperature variable and the ARC was also sig-nificant and negative (β = −0.99, 95% CI −1.66 to −0.32, p = 0.004), indicating that increas-ing temperatures are linked to a slower increase or even a decrease in DALY rates over time. The determination coefficients (R^2^) were small in both (5.5% and 4.3% in the first and second models, respectively).

Additionally, in the regression analysis, when we excluded the countries (n = 18) with annual surface temperatures at or above the 90th percentile (≥ 2.11 °C), the regres-sion coefficients remained statistically significant. The corresponding p-values were 0.013 and 0.045 for the first and second models, respectively.

## 4. Discussion

This present study offers valuable insights into the correlation between mean surface temperatures, dengue DALY rates, and their trends across countries. Our findings indicate a uniform rise in mean surface temperatures across all countries analyzed in 2019 compared to the baseline measurement. These results align with the established scientific consensus on global warming and climate change, highlighting the pressing need to address this urgent issue [18]. The observed temperature increase, with a median of 1.4 °C, underscores the significant change in the Earth’s climate [19]. However, it is important to consider the limitation of conducting an ecological and bivariate analysis when interpreting our findings.

The significant negative relationship between mean surface temperatures and dengue-related DALY rates in 2019 highlights the potential impact of climate change on population health. Higher temperatures were associated with lower DALY rates, suggesting a complex interplay between environmental conditions and disease outcomes. This finding aligns with previous studies linking climate change to various health impacts, including infectious diseases, heat-related illnesses, and exacerbation of chronic conditions [20]. The underlying mechanisms driving this relationship require further investigation to inform targeted interventions and adaptation strategies.

Furthermore, the negative relationship between temperature and the ARC emphasizes the potential mitigating effect of increasing temperatures on the rise of disease burden over time. This relationship was previously described [21]. Countries experiencing higher temperature increases exhibited a slower or even negative ARC, indicating a possible dampening effect of climate change on the upward trajectory of DALY rates; however, it is essential to note that other factors, such as healthcare infrastructure, socio-economic conditions, and public health interventions, can influence these trends and need to be considered in future research.

There are several potential reasons why countries with higher increases in surface temperatures (associated with global warming) may have smaller dengue-related impacts. These reasons may include, among others, the ecology of the vector, seasonality, and climate patterns.

Higher temperatures can affect the ecology and behavior of *Ae.* mosquitoes. The preferred temperature range for *Ae. aegypti* mosquitoes falls between 15 and 35 °C [22]. Deviations from this range can lead to decreased survival rates, reduced reproductive capacity, and alterations in the duration of developmental stages (such as eggs, larvae, and pupae) [23]. Since their surroundings entirely influence the body temperature of mosquitoes, they are highly vulnerable to different forms of thermal stress [24]. Such stress can be induced by the rising average temperatures associated with climate change and extreme climatic events such as heat spikes, which are anticipated to occur more frequently due to increased climate variability [25]. These may lead to reduced mosquito populations and, subsequently, lower dengue transmission.

Furthermore, dengue transmission is impacted by seasonal fluctuations, precipitation patterns, and humidity levels. Climate change can modify these patterns, thereby influencing the breeding, survival, and behavior of *Ae.* mosquitoes [26]. In certain areas, elevated temperatures may induce alterations in rainfall patterns, including heightened frequency or intensity of rainfall events [27]. Such changes can contribute to the proliferation of mosquito breeding sites. Nevertheless, they may also result in the more efficient elimination of larval habitats, subsequently decreasing the overall mosquito population and alleviating the dengue burden.

It is important to note that the impact of global warming on dengue transmission is still an active area of research and the specific effects can vary depending on local conditions and geographical factors; therefore, while these explanations provide a scientific perspective, further studies are necessary to fully understand the complex dynamics between climate change and the dengue burden.

Many other factors that were not included in our analysis may be contributing, at least partially, to the global decrease in dengue burden that we have documented. These factors could encompass intensified vector control measures, such as the elimination of breeding sites [28] and community engagement in prevention activities [29], improved healthcare infrastructure [30], effective urban planning [31], improved sanitation infrastructure [32], and proper waste management [33]. It is important to note that the relative contribution of these factors may vary across different countries and regions.

This study’s strengths include the comprehensive analysis of many countries and the use of DALY rates as a measure of disease burden; however, several limitations should be acknowledged. Firstly, the analysis focused on mean surface temperatures and DALY rates, overlooking other potential climate variables and health indicators. It is crucial to consider that global warming has extensive implications for various environmental conditions. These encompass modified precipitation patterns, rising sea levels, and the occurrence of extreme weather events, among others. Consequently, the climate change variable in our models may encompass and reflect other environmental conditions that impact dengue transmission [34]. Secondly, the analysis of data relies on the quality of the epidemiological surveillance systems in each country; consequently, the burden of dengue may be underestimated, especially in regions with lower income levels.

Additionally, the study design was cross-sectional, limiting our ability to establish causal relationships between temperature, DALY rates, and their trends. Future research should consider longitudinal studies and explore additional climate and health indicators to understand these complex associations comprehensively.

## 5. Conclusions

This study highlights the global increase in mean surface temperatures and the heterogeneous distribution of DALY rates across countries, underscoring the potential impact of climate change on population health outcomes. The observed negative relationship between temperature and DALY rates suggests that as temperatures continue to rise, the burden of disease and disability may decrease; however, it is essential to acknowledge that this pattern may not be universally applicable to all countries, and there may be an unspecified number of countries experiencing increasing trends.

The complex interplay between climate change and health outcomes necessitates a multifaceted approach to mitigate the potential risks. Targeted interventions should be developed to address the specific health concerns of vulnerable populations and regions most susceptible to climate-related health hazards. Additionally, early warning systems and adaptive strategies should be implemented to anticipate and respond to the changing patterns of infectious diseases, heat waves, and extreme weather events.

The findings of this study provide valuable insights for policymakers, public health practitioners, and researchers in understanding the potential consequences of climate change on population health. Continued research in this field is essential for improving our understanding of the mechanisms through which climate change impacts health and for developing evidence-based strategies to protect and promote population well-being in the face of a changing climate.

## Data Availability

Two publicly available datasets were analyzed in this study, both of which were consulted on 25 May 2023. The first dataset was obtained from the Climate Change Dashboard provided by the International Monetary Fund. It contained annual surface temperature change data spanning from 1961 to 2021 and can be accessed at: https://opendata.arcgis.com/datasets/4063314923d74187be9596f10d034914_0.csv. Additionally, we utilized the Global Burden of Disease and Risk Factors Study 2019 (GBD 2019) to obtain data on the burden of dengue. The relevant dataset can be accessed at: https://vizhub.healthdata.org/gbd-results/.

## References

[1] Roy S.K., Bhattacharjee S. (2021). Dengue virus: Epidemiology, biology, and disease aetiology. Can. J. Microbiol..

[2] Du M., Jing W., Liu M., Liu J. (2021). The Global Trends and Regional Differences in Incidence of Dengue Infection from 1990 to 2019: An Analysis from the Global Burden of Disease Study 2019. Infect. Dis. Ther..

[3] Tian N., Zheng J.X., Guo Z.Y., Li L.H., Xia S., Lv S., Zhou X.N. (2022). Dengue Incidence Trends and Its Burden in Major Endemic Regions from 1990 to 2019. Trop. Med. Infect. Dis..

[4] Tran B.L., Tseng W.C., Chen C.C., Liao S.Y. (2020). Estimating the Threshold Effects of Climate on Dengue: A Case Study of Taiwan. Int. J. Environ. Res. Public Health.

[5] Li C., Liu Z., Li W., Lin Y., Hou L., Niu S., Xing Y., Huang J., Chen Y., Zhang S. (2023). Projecting future risk of dengue related to hydrometeorological conditions in mainland China under climate change scenarios: A modelling study. Lancet Planet. Health.

[6] Li C., Lu Y., Liu J., Wu X. (2018). Climate change and dengue fever transmission in China: Evidences and challenges. Sci. Total Environ..

[7] Soneja S., Tsarouchi G., Lumbroso D., Tung D.K. (2021). A Review of Dengue’s Historical and Future Health Risk from a Changing Climate. Curr. Environ. Health Rep..

[8] Chophel Y. (2022). Global Warming and Climate Change (GWCC) Realities. The Nature, Causes, Effects and Mitigation of Climate Change on the Environment.

[9] Al-Ghussain L. (2019). Global warming: Review on driving forces and mitigation. Environ. Prog. Sustain. Energy.

[10] Yoro K.O., Daramola M.O. (2020). CO_2_ emission sources, greenhouse gases, and the global warming effect. Advances in Carbon Capture.

[11] Shakoor A., Ashraf F., Shakoor S., Mustafa A., Rehman A., Altaf M.M. (2020). Biogeochemical transformation of greenhouse gas emissions from terrestrial to atmospheric environment and potential feedback to climate forcing. Environ. Sci. Pollut. Res..

[12] Rahman M.S., Overgaard H.J., Pientong C., Mayxay M., Ekalaksananan T., Aromseree S., Phanthanawiboon S., Zafar S., Shipin O., Paul R.E. (2021). Knowledge, attitudes, and practices on climate change and dengue in Lao People’s Democratic Republic and Thailand. Environ. Res..

[13] GBD 2019 Risk Factors Collaborators (2020). Global burden of 87 risk factors in 204 countries and territories, 1990–2019: A systematic analysis for the Global Burden of Disease Study 2019. Lancet.

[14] Kim Y.E., Jung Y.S., Ock M., Yoon S.J. (2022). DALY Estimation Approaches: Understanding and Using the Incidence-based Approach and the Prevalence-based Approach. J. Prev. Med. Public Health.

[15] International Monetary Found. Climate Change Data: Annual Surface Temperature Change. https://opendata.arcgis.com/datasets/4063314923d74187be9596f10d034914_0.csv.

[16] Global Burden of Disease Collaborative Network. Global Burden of Disease Study 2019 (GBD 2019) Results. https://vizhub.healthdata.org/gbd-results/.

[17] GBD 2019 Risk Factors Collaborators (2022). Global, regional, and national burden of diseases and injuries for adults 70 years and older: Systematic analysis for the Global Burden of Disease 2019 Study. BMJ.

[18] Malla F.A., Mushtaq A., Bandh S.A., Qayoom I., Hoang A.T. (2022). Understanding climate change: Scientific opinion and public perspective. Climate Change: The Social and Scientific Construct.

[19] Abbass K., Qasim M.Z., Song H., Murshed M., Mahmood H., Younis I. (2022). A review of the global climate change impacts, adaptation, and sustainable mitigation measures. Environ. Sci. Pollut. Res..

[20] Al-Marwani S. (2023). Climate change impact on the healthcare provided to patients. Bull. Natl. Res. Cent..

[21] Ware-Gilmore F., Sgro C.M., Xi Z., Dutra H.L.C., Jones M.J., Shea K., Hall M.D., Thomas M.B., McGraw E.A. (2021). Microbes increase thermal sensitivity in the mosquito Aedes aegypti, with the potential to change disease distributions. PLoS Negl. Trop. Dis..

[22] Reinhold J.M., Lazzari C.R., Lahondere C. (2018). Effects of the Environmental Temperature on Aedes aegypti and Aedes albopictus Mosquitoes: A Review. Insects.

[23] Schmidt C.A., Comeau G., Monaghan A.J., Williamson D.J., Ernst K.C. (2018). Effects of desiccation stress on adult female longevity in Aedes aegypti and Ae. albopictus (Diptera: Culicidae): Results of a systematic review and pooled survival analysis. Parasit Vectors.

[24] Bellone R., Failloux A.B. (2020). The Role of Temperature in Shaping Mosquito-Borne Viruses Transmission. Front. Microbiol..

[25] Ornes S. (2018). How does climate change influence extreme weather? Impact attribution research seeks answers. Proc. Natl. Acad. Sci. USA.

[26] Bhatia S., Bansal D., Patil S., Pandya S., Ilyas Q.M., Imran S. (2022). A Retrospective Study of Climate Change Affecting Dengue: Evidences, Challenges and Future Directions. Front. Public Health.

[27] Murray-Tortarolo G.N. (2021). Seven decades of climate change across Mexico. Atmósfera.

[28] Buhler C., Winkler V., Runge-Ranzinger S., Boyce R., Horstick O. (2019). Environmental methods for dengue vector control—A systematic review and meta-analysis. PLoS Negl. Trop. Dis..

[29] Mendoza-Cano O., Hernandez-Suarez C.M., Trujillo X., Ochoa Diaz-Lopez H., Lugo-Radillo A., Espinoza-Gomez F., de la Cruz-Ruiz M., Sanchez-Pina R.A., Murillo-Zamora E. (2017). Cost-Effectiveness of the Strategies to Reduce the Incidence of Dengue in Colima, Mexico. Int. J. Environ. Res. Public Health.

[30] Sujatha C., Sudha R.R., Surendran A.T., Reghukumar A., Valamparampil M.J., Sathyadas I.P., Chandrasekharan P.K. (2021). Social, health system and clinical determinants of fever mortality during an outbreak of dengue fever in Kerala, India. J. Fam. Med. Prim. Care.

[31] Dzul-Manzanilla F., Correa-Morales F., Che-Mendoza A., Palacio-Vargas J., Sanchez-Tejeda G., Gonzalez-Roldan J.F., Lopez-Gatell H., Flores-Suarez A.E., Gomez-Dantes H., Coelho G.E. (2021). Identifying urban hotspots of dengue, chikungunya, and Zika transmission in Mexico to support risk stratification efforts: A spatial analysis. Lancet Planet Health.

[32] Almeida L.S., Cota A.L.S., Rodrigues D.F. (2020). Sanitation, Arboviruses, and Environmental Determinants of Disease: Impacts on urban health. Cienc. Saude Coletiva.

[33] Mahmud M.A.F., Abdul Mutalip M.H., Lodz N.A., Muhammad E.N., Yoep N., Hashim M.H., Paiwai F., Rajarethinam J., Aik J., Muhammad N.A. (2019). Environmental management for dengue control: A systematic review protocol. BMJ Open.

[34] Rocque R.J., Beaudoin C., Ndjaboue R., Cameron L., Poirier-Bergeron L., Poulin-Rheault R.-A., Fallon C., Tricco A.C., Witteman H.O. (2021). Health effects of climate change: An overview of systematic reviews. BMJ Open.

